# The health-promoting functions of γ-aminobutyric acid (GABA) and lactic acid bacteria metabolism: From biosynthesis to applications in the food industry

**DOI:** 10.1016/j.fochx.2026.104627

**Published:** 2026-10-18

**Authors:** Hui Zhou, Wenying Yuan, JiaoJiao Hu, Mengyao Liu, Jie Luo, Baokun Li, Xiankang Fan

**Affiliations:** a College of Food Science and Technology, Hunan Agricultural University, Changsha 410128, China; b Key Laboratory of Geriatric Nutrition and Health (Beijing Technology and Business University), Ministry of Education, China; c Hunan Institute of Metrology and Testing, Changsha 410014, China; d School of Science and Technology, Shihezi University, Shihezi, Xinjiang 832003, China

**Keywords:** GABA, Lactic acid bacteria, Glutamate decarboxylase system, Health-promoting functions

## Abstract

GABA is a principal inhibitory neurotransmitter with systemic regulatory roles. This review synthesizes the health-promoting functions of GABA and the molecular mechanisms of its production in LAB. GABA regulates neuronal excitability via GABA receptors, suppresses inflammation by inhibiting the TLR4/NF-κB pathway, and modulates the gut-brain axis through microbiota remodeling. LAB synthesize GABA primarily via the glutamic acid decarboxylase (GAD) system, with an alternative GABA-T-mediated non-GAD pathway that requires further validation, a process tightly coupled to acid resistance. Optimization strategies include genetic engineering, fermentation control, physical stimulation, and synthetic co-culture. These strains are widely applied in dairy, plant-based beverages, and meat, enhancing flavor while sleep-improving, and microbiota-modulating benefits. However, significant challenges persist, including incomplete substrate conversion causing off-flavors, poor GABA stability in food, and genetic instability during scale-up. This work uniquely integrates non-neuronal GABA functions, metabolic diversity beyond the canonical GAD pathway, and practical challenges of GABA incorporation into food matrices.

## Highlights

• GABA regulates neuronal, immune, and gut-brain signaling in preclinical models.
• LAB synthesize GABA via GAD system; a strain-specific GABA-T route need validation.
• GABA-enriched foods may aid flavor and offer potential health benefits.
• Genetic editing, co-culture and stimulation may boost GABA yield for scale-up.
• Incomplete conversion, matrix instability & genetic instability are key bottlenecks.

## Graphical abstract

**Figure:**
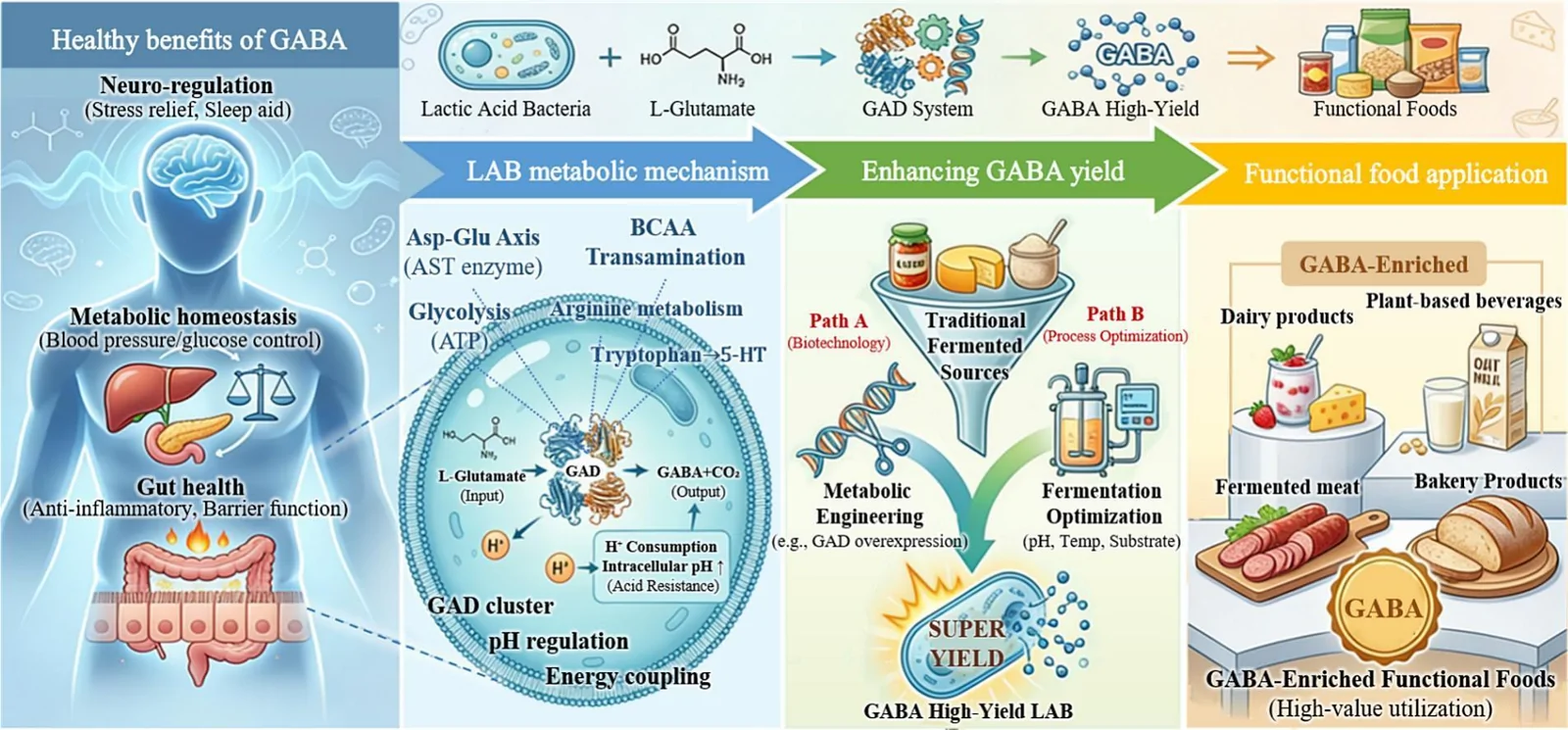

## 1 Introduction

γ-Aminobutyric acid (GABA), a four‑carbon non-protein amino acid, is the main inhibitory neurotransmitter in the mammalian central nervous system. It plays a key role in regulating neuronal excitability, reducing anxiety, and promoting sleep (Xia et al., 2021; Zhang et al., 2021). The biological significance of GABA has expanded far beyond these classical neurotransmitter roles. Contemporary research now recognizes it as an endogenous bioactive metabolite with pleiotropic systemic regulatory functions extending well past the central nervous system (Belelli et al., 2022; Xie et al., 2025). A growing body of evidence links GABA to the pathophysiology of mood disorders such as major depressive disorder. This link is thought to arise from GABA-mediated modulation of excitatory-inhibitory balance and bidirectional gut-brain axis signaling (Godfrey et al., 2025; Lissemore et al., 2018; Zhao et al., 2025). It also orchestrates glucose and lipid metabolism across multiple organs, conferring potential benefits for metabolic homeostasis (Jiang et al., 2024). GABA exerts pronounced anti-inflammatory effects by modulating immune responses and has been associated with reconfiguring gut microbiota composition in preclinical studies (Chen et al., 2024). This paradigm shift has recast GABA from a neural signaling molecule into a multifunctional systemic regulator, substantially broadening its therapeutic and nutraceutical applications. GABA is now viewed as a promising bioactive compound for functional foods that target neurological, metabolic, and immunomodulatory health.

In light of the broad physiological significance of GABA, dietary supplementation through functional foods represents an attractive strategy. However, most natural food matrices contain endogenous GABA at levels too low to produce physiologically relevant therapeutic effects. This limitation calls for efficient enrichment strategies or de novo biosynthesis approaches. GABA synthesis in food is primarily achieved through plant enrichment, chemical synthesis, and enzymatic conversion (Sun et al., 2022). These conventional approaches suffer from low catalytic efficiency, potential safety concerns associated with chemical residues, or prohibitive production costs. By contrast, microbial fermentation offers integrated advantages in terms of biosafety, catalytic efficiency, and cost-effectiveness (Xiao et al., 2025). GABA is directly produced by selected strains through a natural, scalable process (Hou et al., 2024), which has made this approach the predominant technical route for GABA production in the food industry. Among the microorganisms used, lactic acid bacteria (LAB) are considered ideal biological carriers for synthesizing food-grade GABA. This is largely because they hold generally recognized as safe (GRAS) status and have a long history of use in traditional fermented foods (Fitsum et al., 2025). The toxicological safety of GABA as a food ingredient has been rigorously established through comprehensive subchronic toxicity studies. A 13-week oral toxicity evaluation in rats administered GABA at doses up to 2500 mg/kg body weight/day demonstrated no test substance-related mortality or toxicologically significant alterations in clinical pathology, organ weights, or histopathological examinations (Takeshima et al., 2014). However, as the principal inhibitory neurotransmitter, GABA exerts dose-dependent pharmacological effects on the central nervous system. Excessive intake may theoretically potentiate central nervous system (CNS) depression. This could lead to enhanced fatigue, drowsiness, or reduced cognitive alertness. The risk is particularly notable when GABA is co-administered with other GABAergic agents such as alcohol, benzodiazepines, or antihypertensive medications (Oketch-Rabah et al., 2021). International regulatory frameworks exhibit considerable heterogeneity. Health Canada recommends a daily intake of 50 to 3000 mg, not exceeding 750 mg per single dose, while China's national standard specifies a maximum daily intake of 500 mg for GABA as a new food resource. Within these established safety limits, GABA is considered safe for the general population. However, susceptible subgroups such as pregnant women, paediatric populations, and individuals with compromised hepatic function require heightened vigilance. Notably, the fermentation conditions that favor GABA biosynthesis, including elevated amino acid substrate availability and acidic pH, can concomitantly promote biogenic amine formation (e.g., histamine, tyramine) through non-GAD amino acid decarboxylase activities in certain LAB strains (Spano et al., 2010). This represents a critical food safety dimension that necessitates integrated risk assessment alongside functional optimization (Gardini et al., 2016). For instance, we previously found that *L. brevis* J1, isolated from fish intestine, could produce up to 241.30 μg/mL of GABA in adzuki bean sprout fermented milk (Wu et al., 2021). LAB synthesize GABA primarily through the glutamate decarboxylase system, which catalyzes the decarboxylation of L-glutamate to form GABA (Liu et al., 2023). This reaction consumes intracellular protons, thereby linking the maintenance of intracellular pH homeostasis with product synthesis. Key factors include environmental pH, the cofactor pyridoxal 5′-phosphate (PLP), and a multi-level transcriptional regulatory network (Qin et al., 2024). Xie et al. (2023) also identified a putative GABA-T-mediated pathway in GAD-deficient *Lentilactobacillus curieae*, suggesting that alternative routes may operate under specific genetic backgrounds. These findings highlight strain-level metabolic diversity within LAB, opening up new avenues for strain screening, fermentation optimization strategies and the engineering of bacterial strains.

GABA-enriched foods demonstrate the versatility of GABA-producing LAB. For example, GABA-rich yogurt was developed by Fan, Jiang, et al. (2023) and Fan, Yu, et al. (2023) using *Levilactobacillus brevis* CGMCC 1.5954, achieving a GABA content of 147.36 mg/100 mL. Anxiety and memory decline were significantly alleviated in mice with circadian rhythm disorders via the gut-brain axis (Fan et al., 2025). Hong Le et al. (2021) released free glutamic acid (Glu) in soymilk through enzymatic hydrolysis using Alcalase and Flavourzyme, then converted it to GABA via fermentation with *L. plantarum* LMG6907. Despite these successes, significant challenges persist. Incomplete conversion of the precursor monosodium glutamate (MSG) leads to potential off-flavors and consumer concerns (Banerjee et al., 2021).Complex microbial metabolic activities frequently produce undesirable organoleptic attributes (off-tastes and textural defects) in the final product. Moreover, the suboptimal storage stability of GABA in complex food matrices results in progressive loss of functional bioactivity over time (Ma, Wang, Yang, et al., 2022; Ma, Wang, Zhou, et al., 2022; Zhang et al., 2024). GABA stability in foods is influenced by processing conditions, storage parameters, and interactions with food components that affect both its measurable concentration and bioavailability. Microencapsulation technology (especially when combined with probiotics) has been proven to be a very effective and universal strategy for enhancing the stability of GABA during processing and storage (Misra et al., 2025). However, rigorous validation through well-designed clinical trials remains necessary, to establish the actual health benefits and underlying mechanisms of GABA derived from such products in human subjects. GABA concentration in a fermented food does not by itself guarantee a meaningful physiological outcome. The ultimate health effect depends on the dose consumed, the bioavailability of GABA from the food matrix, its stability during gastrointestinal transit, and absorption efficiency (Nakatani et al., 2022; Venturi et al., 2019). Food matrices can influence these parameters through interactions with components that may protect or hinder GABA delivery. Moreover, the relationship between dietary GABA intake and physiological responses remains incompletely understood, and effective doses in preclinical studies often exceed those achievable through normal consumption of GABA-enriched foods. These considerations highlight the need for future research on the pharmacokinetics and pharmacodynamics of food-derived GABA in humans.

This narrative review systematically reviews the latest developments and existing challenges regarding the physiological functions of GABA, the biosynthetic mechanisms in LAB, and its applications in various food products. Literature was retrieved from PubMed and Web of Science, with emphasis on peer-reviewed studies published within the last decade and particularly recent advances in the past five years. Previous reviews have examined strain screening and fermentation optimization (Pannerchelvan et al., 2023), postbiotic activity along the gut-brain axis (Braga et al., 2024), and dietary sources with enrichment technologies. This review brings together three topics that have rarely been discussed together. It explores non-neuronal GABA roles in metabolic and immune regulation. It also covers metabolic diversity beyond the canonical GAD pathway, including the GABA-T-mediated route. Finally, it addresses practical hurdles in adding GABA to food matrices, including precursor conversion, sensory quality, storage stability, and microencapsulation. These insights would help researchers develop GABA-enriched functional foods and design better nutritional strategies for mood disorders and metabolic health.

## 2 Physiological functions and mechanisms of GABA

### 2.1 Regulation of nervous system functions

GABA is the principal inhibitory neurotransmitter in the central nervous system. It regulates neuronal excitability and synaptic transmission through involving ionotropic GABA_A_ receptors and metabotropic GABA_B_ receptors (Li et al., 2017). Disruptions in this excitation-inhibition balance have been associated with anxiety, depression, epilepsy, and neurodegenerative diseases (Roy et al., 2018). Recent research has revealed multimodal functional regulation of the GABAergic system (Table 1). Several intervention strategies effectively increase GABA concentration in key brain regions, including oral GABA supplementation (Xu, Ge, et al., 2025), regular exercise (Cao et al., 2021), and targeted cranial ultrasound neuromodulation (Phan et al., 2025). These approaches alleviate anxiety-like behaviors (Cui et al., 2020), inhibit refractory epileptic seizures, and promote axonal regeneration following spinal cord injury (Barreiro-Iglesias et al., 2019).

**Table 1:** Researches on GABA functions and the GABAergic system.

Experiment object	Mechanism	Effect of regulation	References
Simulated human colon	Strengthening intestinal epithelial barrier by increasing tight junction protein concentration and positively regulating gut microbiota	Increasing abundance of beneficial gut microbes (e.g., *Lactobacillus* and *Bifidobacterium*) and reducing *Enterococcus* and *Streptococcus* numbers	(Kaur et al., 2023)
Rats	Increasing GABA levels with bergamot essential oil (BEO) and AlCl3	Alleviating anxiety-like behaviors in aluminum-treated animals	(Cui et al., 2020)
	Lipid metabolism regulation, glucose homeostasis improvement, and antioxidant effects	Improving metabolic abnormalities induced by a high-fat diet in rats	(Shang et al., 2018)
	Producing GABA via GadB - catalyzed decarboxylation of glutamate	Modulating sensory neuronal activity in a rat fecal retention model of visceral hypersensitivity	(Pokusaeva et al., 2017)
	Reducing harmful gut microbiota and regulating striatal GABA-glutamate metabolism to alleviate TS-like behaviors	Relieving Tourette - like behaviors	(Xu, Ge, et al., 2025; Xu, Li, et al., 2025)
Pig	Increasing PPARγ and GABARs mRNA expression, and decreasing TLR4/NF-κB signaling expression	Alleviating LPS-induced liver inflammation	(Zhang, Zhao, et al., 2022)
Mice	Decreasing mRNA and protein expression of GAD65, GAD67, and VGAT	Anxiety – and depression - like behaviors in fluoride-poisoned mice	(Cao et al., 2021)
	Modulating gut microbiota	Improving fructose-induced hepatic steatosis	(Chen et al., 2024)
	Improving systemic insulin resistance and insulin-dependent glucose uptake in subcutaneous inguinal fat tissue	Inhibiting subcutaneous fat inflammation in obese patients	(Hwang et al., 2019)
	Activating the PI3K/Akt pathway to promote muscle synthesis and reduce muscle degradation	Age-related obesity and sarcopenia induced by a high-fat diet	(Jin et al., 2023)
	Specific therapeutic regulation of GABAergic neurotransmission and inflammatory pathways	Enhancing neuronal plasticity to promote stroke recovery	(Michalettos & Ruscher, 2022)
	The monoamine oxidase B pathway of putrescine in reactive astrocytes	Playing a role in Alzheimer ‘s-related memory deficits and sex - related differences	(Roy et al., 2018)
	Inhibiting IL-1β production by inflammatory macrophages	Alleviating LPS-induced sepsis and high-fat diet-induced obesity in mice	(Fu et al., 2022)
Lamprey	Acting through GABAB receptors and GABAA receptors	Neuroprotection and regeneration promotion after spinal cord injury	(Barreiro - Iglesias et al., 2019)
Juvenile crabs	Sphingolipid signaling pathway and arachidonic acid metabolism pathway	Inflammation regulation and neuroprotection	(Zhang, Guo, et al., 2023; Zhang, Liu, et al., 2023)
Goldfish	Inhibiting the increase in circulating growth hormone levels stimulated by estradiol	Inhibiting growth hormone release	(Trudeau et al., 2000)

GABA transcends its classical neurotransmitter function, operating as a systemic integrator that orchestrates the neuro-immune-metabolic axis (Fig. 1). It exerts multi-system effects through CNS inhibitory transmission, metabolic regulation (insulin secretion, hepatic protection), and potential gut-brain axis functionalities that have been associated with microbiota remodeling and epithelial barrier integrity in preclinical studies (W. Zhang et al., 2021; Ferreira et al., 2025; Wang, Chen, et al., 2025). These regulatory considerations point toward a clear mechanism for using GABA-enriched LAB-fermented foods to address sleep and stress-related disorders. However, the multi-target effects observed in fermented matrices could stem from GABA itself or from other bioactive compounds generated during fermentation. This uncertainty persists because most evidence derives from purified GABA or non-fermented preparations. An important conceptual challenge in interpreting these findings is that health effects associated with GABA-producing LAB or GABA-enriched foods may arise from multiple sources, including viable LAB cells, GABA produced during fermentation, other microbial metabolites, or synergistic interactions among these components and the food matrix.

**Fig. 1:**
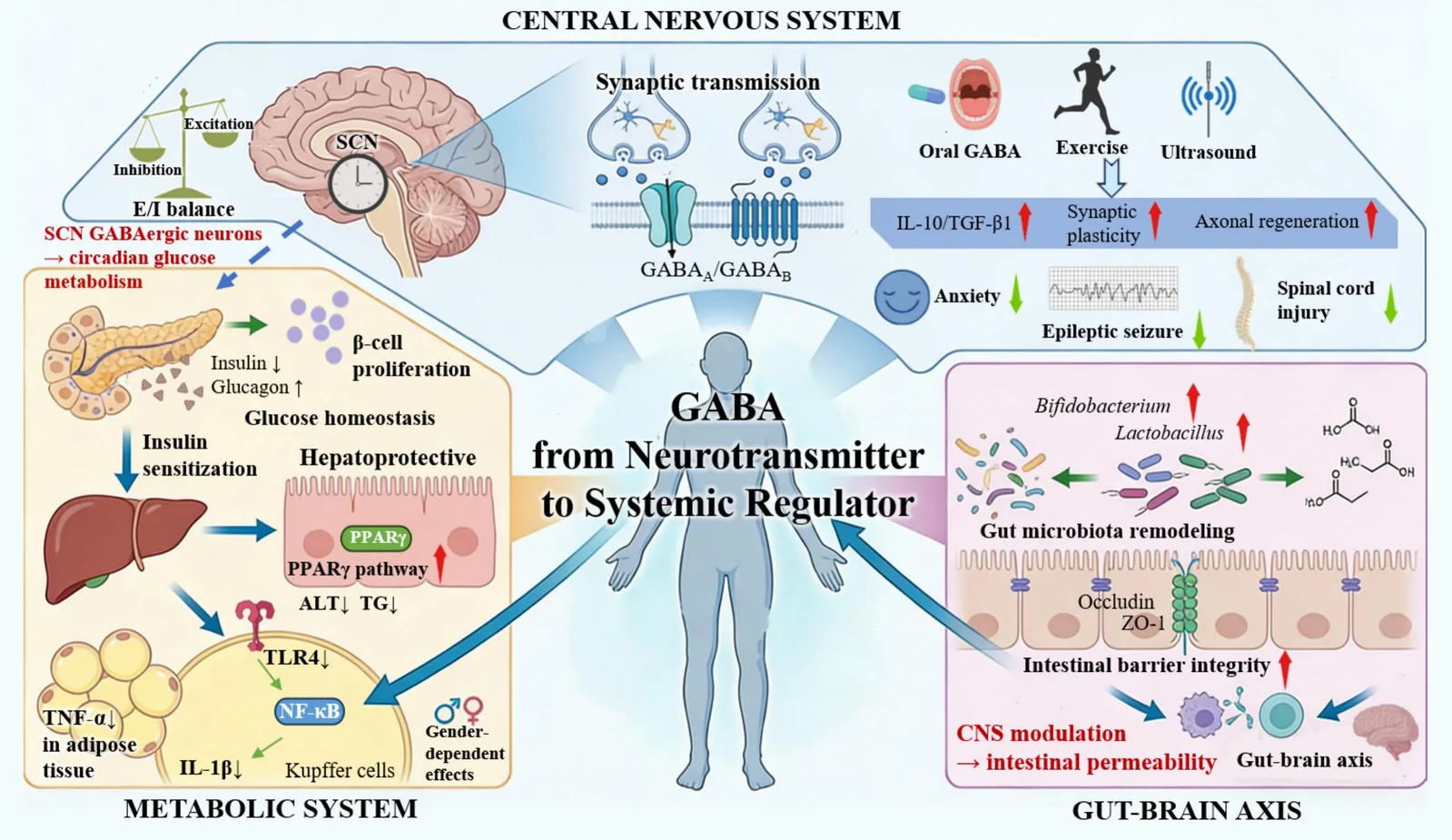
GABA links nervous, metabolic, and immune systems. CNS balance is maintained via GABA _A/B_ receptors; anxiety/epilepsy/SCI are alleviated by oral GABA, exercise, or ultrasound. Metabolism is regulated via insulin/glucagon secretion, PPARγ, and TLR4/NF-κB. Gut-brain axis remodeling has been associated with enrichment of probiotics, upregulation of tight junctions, and modulation of CNS function via gut permeability in preclinical studies.

In a chronic restraint stress mouse model, oral GABA (20 mg/kg) raised prefrontal cortex GABA levels, reversed stress-induced complement system dysregulation, and upregulated IL-10 and TGF-β1 expression (Xu, Ge, et al., 2025; Xu, Li, et al., 2025). These findings suggest that anti-anxiety effects involve both classical GABAergic neurotransmission and neuro-immune interactions. High-GABA jujube extract obtained through LAB fermentation regulates glutamate/GABA balance and enhances GABA_A_ receptor expression in a caffeine-induced insomnia model, effects blocked by GABA_B_ antagonists (Bae et al., 2023). In a recent study, GABA from *Lactococcus lactis* ZFM559 (pGABA, 99.47% purity) relieved depression-like behaviors (Han et al., 2025). The treatment suppressed HPA axis hyperactivity, reduced inflammation, and modulated serotonergic metabolism. These results align with evidence that GABAergic signaling dampens HPA axis hyperactivity by inhibiting corticotropin-releasing factor neurons (Tette et al., 2022). GABAergic signaling is also widely present in invertebrate peripheral nervous systems, indicating a fundamental role in stress adaptation across organisms (Lv et al., 2023). In neurodegenerative and injury-related contexts, GABA's neuroprotective effects are increasingly recognized through diverse mechanistic pathways. Cao et al. (2021) examined a rat model of chronic fluoride neurotoxicity. Reduced expression of GAD65, GAD67, and VGAT was linked to anxiety- and depression-like behaviors. These findings point to impaired GABA synthesis and vesicular transport as a key disease mechanism. GABAergic mechanisms also contribute to neuroprotection through sphingolipid signaling and arachidonic acid metabolic pathways (Zhang, Liu, et al., 2023). These cross-species findings highlight the phylogenetic conservation of GABAergic functionalities in neuronal protection and stress adaptation.

Several caveats should be considered when translating preclinical GABAergic research into clinical use, as the effects of GABA are highly context-dependent. In depression, baseline GABA and glutamate-to-GABA ratios are poor predictors of antidepressant response, which suggests that efficacy depends more on the neural microenvironment than on absolute neurotransmitter levels (Dai et al., 2025; Sarawagi et al., 2021). Translation from animal models to humans also faces substantial constraints, since blood-brain barrier permeability, species differences, and individual heterogeneity all limit direct extrapolation, and factors such as gender effects and baseline anxiety add further complexity (Roy et al., 2018; Zhang, Kitaori, & Lai, 2025). Fermented foods present additional challenges because they contain other bioactive metabolites such as organic acids and peptides, and this makes it difficult to attribute observed effects to GABA alone. Moreover, the bioavailability of GABA from food matrices after digestion remains unclear. Future work should move beyond measuring total GABA levels to investigate receptor subtype-specific functions and cross-talk with other neurotransmitter systems.

### 2.2 Regulation of metabolic homeostasis

GABA helps regulate metabolism through coordinated action across multiple organs. It links central nervous system inhibition with the gut-brain axis through shared molecular pathways. These include PPARγ signaling, the TLR4/NF-κB pathway, and regulation of insulin and glucagon secretion (Fig. 1). GABAergic neurons in the suprachiasmatic nucleus regulate the circadian rhythm of insulin-mediated hepatic glucose production inhibition, linking central GABAergic activity with hepatic glucose metabolism (Feng et al., 2022). This central-peripheral crosstalk connects hypothalamic GABAergic tone to peripheral metabolic organs through neurohumoral signaling pathways. Hepatic metabolic status can reciprocally modulate central GABAergic activity through circulating metabolites and inflammatory mediators. In pancreatic tissue, GABA promotes β-cell proliferation and modulates insulin and glucagon secretion balance, thereby improving blood glucose homeostasis in diabetic mice (Sabna et al., 2025). Combined administration of Ang-(1–7) and GABA inhibits β-cell dedifferentiation, demonstrating synergistic therapeutic potential (Ma et al., 2020). Endogenous β-cell-derived GABA is critical for modulating insulin secretion by maintaining proper Ca^2+^ oscillation dynamics through GABA_A_ and GABA_B_ receptor signaling (Ferreira et al., 2025). GABA deficiency causes excessive insulin secretion and defective Ca^2+^ oscillations, while high-fat diet-fed mouse islets and human type 2 diabetic islets exhibit unresponsiveness to GABA with impaired Ca^2+^ oscillations (Hagan et al., 2022). These findings reveal that GABA is essential for β-cell function, but metabolic disease induces GABA resistance, creating a vicious cycle of progressive islet dysfunction. GABA-enriched functional foods therefore likely serve better as pre-diabetic prevention than as therapy for established diabetes, unless combined with strategies that restore GABA receptor sensitivity, such as anti-inflammatory or fiber-rich fermentation approaches.

Regarding hepatic metabolism, GABA shows significant insulin-sensitizing and hepatoprotective effects. GABA-fortified wheat bran reverses high-fat diet-induced insulin resistance in rats through hepatic PPARγ pathway activation and oxidative stress inhibition (Shang et al., 2018). GABA reduces serum ALT levels by 57% and hepatic triglyceride content by 39% in fructose-induced fatty liver mice (Chen et al., 2024). PPARγ activation suppresses de novo lipogenesis and enhances fatty acid oxidation. Gut microbiota restructuring simultaneously increases Lactobacillus abundance and butyrate production. These microbial changes then reinforce PPARγ signaling and strengthen intestinal barrier integrity (Wang et al., 2021). This establishes a self-amplifying metabolic-gut feedback loop wherein hepatic and gut-derived signals mutually reinforce homeostatic maintenance, providing a novel molecular connection for microbiota-mediated metabolic regulation. Cui, Yang, et al. (2025) confirmed that oral GABA treatment ameliorates high-fat diet-induced hepatic insulin resistance through suppression of Kupffer cell activation and TLR4/NF-κB pathway inhibition. GABA evokes membrane currents and hyperpolarizes macrophages, thereby decreasing palmitate-induced Ca^2+^ influx and TNF-α production (Tsomidis et al., 2026), establishing the cellular mechanism by which GABA suppresses hepatic inflammation and improves insulin sensitivity.

Beyond glucose and lipid metabolism, GABA is involved in bone metabolism and stress energy balance regulation. GABA effectively antagonizes glucocorticoid-induced osteoporosis in zebrafish models (Arachchilage Hasitha Maduranga Karunarathne et al., 2023). GABA upregulates bone formation-related genes including BMP-2, SPARC, and calcitonin receptor through GABA receptor activation (Muhammad et al., 2013), though GABA_B_ receptor activation inhibits osteoblast differentiation markers in murine calvarial cells (Takahata et al., 2011). These apparently contradictory findings indicate that GABA's effects on bone metabolism are receptor subtype- and context-dependent, cautioning against the indiscriminate generalization of bone health benefits. Under stress conditions, GABA supplementation optimizes energy utilization efficiency and enhances organism stress tolerance by reducing glucose and lactate accumulation in hypoxic fish (Varghese et al., 2020; Yu et al., 2024). GABA's metabolic effects exhibit gender dependence, significantly promoting growth hormone-releasing peptide expression only in male mouse stomach (Begazo-Jimenez et al., 2025), highlighting the necessity of considering gender factors in research and application. GABA has been reported to influence gut microbial composition in a strain-specific manner, promoting beneficial bacteria such as *Lactobacillus* and *Bifidobacterium* while inhibiting pathogenic species (Chen et al., 2024). These observations suggest that gut microbiota restructuring may serve as a proximal trigger that propagates metabolic benefits distally to the liver, pancreas, and adipose tissue. This interaction enhances gut barrier function and regulates short-chain fatty acid metabolism, thereby achieving systemic metabolic regulation.

### 2.3 Anti-inflammatory and barrier functions

GABA exhibits anti-inflammatory and barrier protective functions in peripheral tissues through multi-target and multi-organ interactions. In macrophages and Kupffer cells, GABA acts through GABA_A_ and GABA_B_ receptors to suppress TLR4/NF-κB signaling and reduce pro-inflammatory cytokine production including IL-1β and TNF-α (Tsomidis et al., 2026). This effect is mediated by GABA receptor activation rather than direct binding of GABA to TLR4, as GABA does not possess structural features typical of TLR4 ligands (Fu et al., 2022). These findings have been demonstrated in lipopolysaccharide-induced septic murine models and in isolated macrophage cultures. GABA-treated mesenchymal stem cells reduce pro-inflammatory TNF-α secretion by approximately 75% (Hwang et al., 2019). TLR4/NF-κB suppression appears to be a conserved anti-inflammatory mechanism of GABA across macrophages, mesenchymal stem cells, and Kupffer cells. This suggests that GABA-enriched fermented products could benefit multiple tissues, provided that bioaccessible concentrations reach physiologically relevant levels. GABA-rich extracts from *Curcuma zedoaria* (Boby et al., 2021) and fermented *Moringa oleifera* leaves (Zheng et al., 2023) effectively reduce pro-inflammatory factor levels in animal models, an effect attributed to GABA receptor-mediated suppression of the TLR-4/NF-κB inflammatory signaling pathway. These plant and fermentation-derived sources appear to engage similar inflammatory pathways as endogenous GABA, supporting the potential development of naturally sourced GABA-enriched functional foods. In hepatic Kupffer cells, GABA receptor activation inhibits the TLR4 pathway, thereby improving liver inflammatory status (Zhang, Zhao, et al., 2022). In gastric tissue, GABA increases superoxide dismutase and catalase activities while reducing malondialdehyde content, achieving antioxidant and anti-inflammatory effects (Xie et al., 2017). This suggests that GABA-enriched functional foods can be strategically targeted to the dominant pathology of each organ, with multi-system benefits propagating through shared signaling nodes such as NF-κB and Nrf2. Regarding barrier protection, GABA upregulates intestinal epithelial tight junction proteins such as occludin and ZO-1, and improves intestinal permeability by modulating the glutamate/GABA balance in the central nervous system (Xu, Li, et al., 2025). This gut-brain bidirectional regulatory mechanism explains the potential therapeutic efficacy of GABA in neuroimmune diseases and operationalizes the “gut-brain axis” hypothesis at the level of specific molecular interactions (Kaur et al., 2023).

In a dexamethasone-induced oxidative stress and inflammation model in broiler chickens, dietary GABA at 100 mg/kg and 200 mg/kg significantly modulated adverse effects on growth parameters (Fathi et al., 2023). It reduced dexamethasone-induced elevation of serum IL-6 and IL-10, enhanced antioxidant enzyme activities, decreased malondialdehyde content, and improved lipid profiles. These findings indicate comprehensive anti-inflammatory and antioxidant effects in this poultry model. In a mouse model of dextran sodium sulfate-induced colitis, oral GABA at 40 mg/kg for seven days significantly attenuated disease severity (Gold & Loeza-Alcocer, 2024). GABA administration reduced colon permeability, disease activity index, and histological score. The treatment also prevented body weight loss and colon shortening while suppressing pro-inflammatory cytokines such as TNF-α, IFN-γ, and IL-12. Expression of the anti-inflammatory cytokine IL-10 simultaneously increased in colon tissue. The overall response pattern observed in this mouse model supports further investigation into the development of GABA-enriched fermented foods for inflammatory bowel conditions. Nevertheless, translation to human disease still requires validation of dose-exposure relationships and matrix-specific bioaccessibility. In snakehead fish, GABA at 60, 90, and 120 mg/kg for 56 days demonstrated dose-dependent improvement of intestinal mucosal barrier function (Wu et al., 2025. The treatment significantly reduced Evans blue dye leakage and colonic pathological changes. GABA also upregulated tight junction proteins such as occludin, claudin-4, and ZO-1 while inhibiting excessive autophagy in intestinal epithelial cells. Both protective actions observed in this fish model particularly relevant for fermented food applications, since processing and storage stresses can compromise epithelial integrity through multiple pathways.

Research has revealed complexity and contradictions in GABA's anti-inflammatory and barrier functions. Some studies found that GABA treatment may increase susceptibility to dextran sodium sulfate-induced colitis (Wang, Wang, Nan, et al., 2024; Wang, Wang, Wang, et al., 2024; Zhang, Wang, et al., 2022). In contrast, lower GABA content in lychee fermentation products showed stronger protective effects on colon tissue (Huang et al., 2024). These contradictions may reflect hormesis (biphasic dose-response), matrix synergy with other bioactives (e.g., polyphenols), or species-specific receptor differences, which are not mutually exclusive. GABA's immune effects are context-dependent, varying with dose, tissue milieu, metabolites, and disease stage, rather than being inherently pro- or anti-inflammatory (Shi, 2022). Stratifying these factors is therefore critical for safe GABA-enriched food development. The fermentation conditions favoring GABA production also promote biogenic amine formation in certain strains, with histamine and tyramine accumulation potentially undermining the antihypertensive effects observed in preclinical studies.

## 3 Mechanisms of GABA metabolism by LAB

### 3.1 Molecular mechanisms and regulation of the GAD system

The GAD system is a conserved acid resistance mechanism widely distributed in bacteria, particularly in lactic acid bacteria (Lu et al., 2025). GadA and GadB are PLP-dependent glutamate decarboxylases that convert L-glutamate to GABA through α-decarboxylation. GadC functions as a glutamate/GABA antiporter, handling electrogenic substrate uptake and product efflux. Additional regulatory machinery detects extracellular pH and triggers transcriptional reprogramming. The GAD system operates as a multi-level regulatory network that integrates environmental pH signals, substrate availability, and cofactor supply to coordinate GABA production with cellular acid tolerance. Microbial production has become the preferred approach for GABA biosynthesis, though the underlying mechanisms still need further investigation (Yogeswara, Kittibunchakul, et al., 2020; Yogeswara, Maneerat, & Haltrich, 2020). The central reaction is catalyzed by GAD, which requires PLP as a cofactor to convert L-glutamate into GABA (Fig. 2). Substrate uptake occurs via the glutamate/GABA antiporter, followed by intracellular decarboxylation by GadA or GadB and subsequent product efflux (Gong et al., 2020). Each decarboxylation event consumes an intracellular proton (H^+^) to alleviate cytoplasmic acidification stress under acidic conditions, establishing a direct functional link between GABA production and the GAD system's acid resistance function. This proton-consuming reaction, coupled with the glutamate/GABA antiport activity of GadC, enables the cell to maintain intracellular pH homeostasis while exporting the synthesized GABA (Li et al., 2022). This acid tolerance mechanism is particularly relevant for LAB survival during fermentation, where organic acid accumulation progressively lowers the environmental pH.

**Fig. 2:**
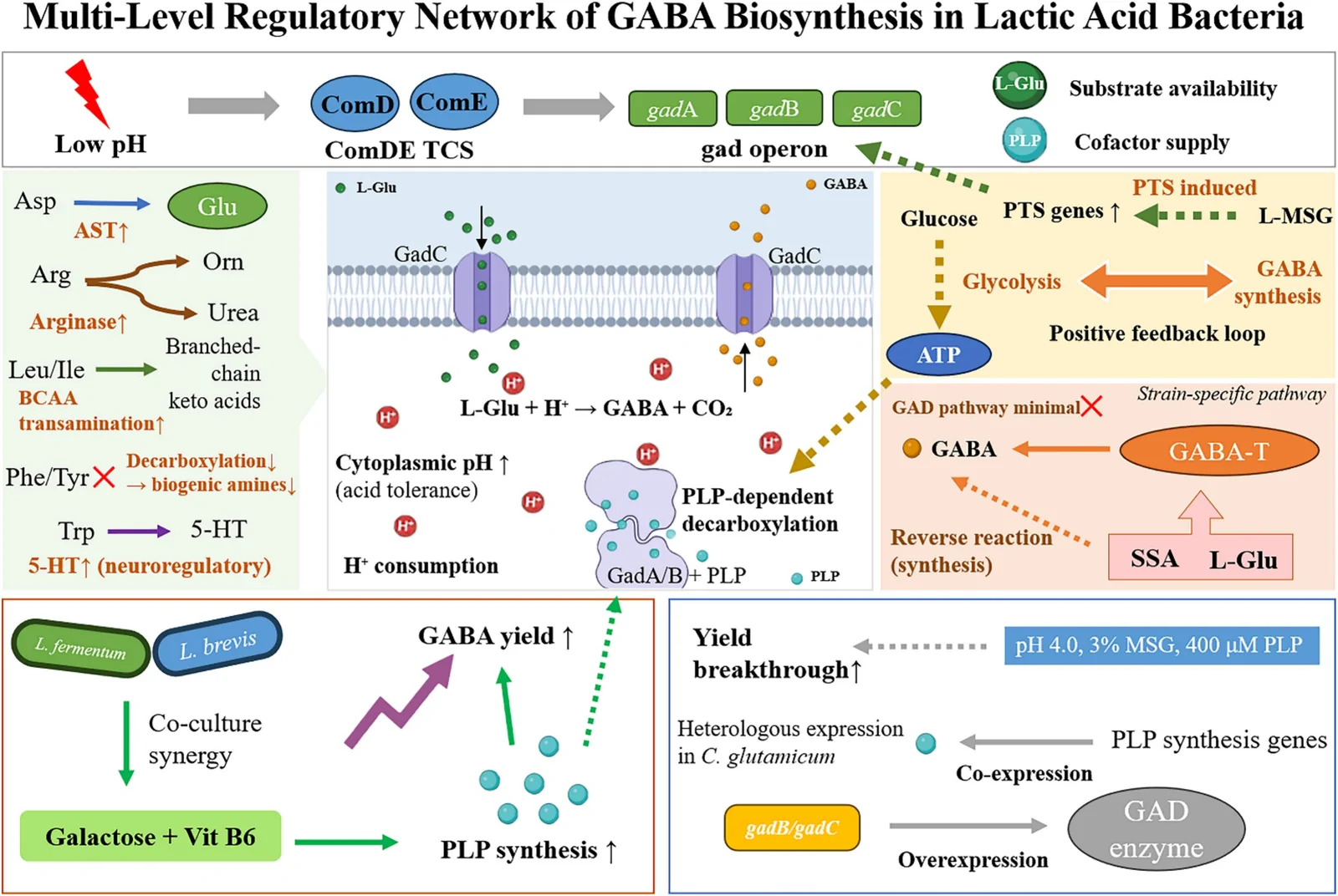
LAB GABA biosynthesis integrates GAD, ComDE, PLP, and GadC. Low pH induces gad operon; substrate/PLP modulate activity; GadC couples tolerance with efflux. Yield is enhanced by co-culture, PLP gene expression, and gadB/C overexpression.

At the transcriptional regulatory level, gad operon expression is primarily induced by acidic pH through two-component signal transduction systems (TCS) such as ComDE (Saint Martin et al., 2023). Beyond pH, multiple environmental and physiological signals modulate GAD system activity. The availability of L-glutamate and its interaction with GadC positively regulate GAD enzyme activity through allosteric effects (Im et al., 2020). Carbon source availability and growth phase dynamics also influence gad operon expression, with carbohydrate limitation and entry into the stationary phase frequently enhancing GABA productivity (Zhu et al., 2020). Stress response regulators likely help fine-tune GAD expression under varying environmental conditions. The general stress sigma factor and transcriptional repressors identified in phylogenetically related bacteria are among these regulators (Ma et al., 2003). However, their precise functions in LAB remain poorly understood. PLP stabilizes the reaction intermediate by forming a Schiff base at the active center, providing the chemical basis for efficient decarboxylation. PLP availability serves as a critical rate-limiting factor for GAD activity, as its supply directly determines the catalytic efficiency of the decarboxylation reaction (Son et al., 2022). Metabolic engineering strategies frequently target the rate-limiting steps within this system to enhance GABA yield. In *Levilactobacillus brevis* LAB6, the complete GAD gene cluster (*gadA, gadB, and gadC*) is the key genetic basis for high GABA yield (Matta et al., 2024). Optimizing fermentation conditions can increase the yield, one effective approach is to maintain pH at 4.0 while supplementing with monosodium glutamate and PLP. Overexpression of *gadB* or *gadC*, or co-expression of genes from the PLP synthesis pathway, has proven effective in overcoming yield limitations of native strains.

Significant strain heterogeneity exists in GABA synthesis pathways among LAB. Although the mainstream pathway relies on the GAD system, alternative pathways should not be overlooked. In the canonical GABA shunt, GABA transaminase (GABA-T, encoded by gabT) catalyzes the conversion of GABA to succinic semialdehyde, which subsequently enters the tricarboxylic acid cycle. This reaction proceeds in the degradative direction and represents the primary physiological function of GABA-T in most microorganisms, where GABA serves predominantly as a nitrogen or carbon source rather than as a biosynthetic intermediate. The reverse reaction, which converts succinic semialdehyde and glutamate to GABA, would require the enzyme to operate in the biosynthetic direction. This direction is thermodynamically unfavorable under physiological conditions and has not been established as a general pathway in LAB (Zhu et al., 2020). Consequently, the net accumulation of GABA in LAB is governed by the flux balance between GAD-mediated synthesis and GABA-T-mediated degradation (Sharafi et al., 2019). Intracellular pH, substrate availability, and transport dynamics collectively influence this equilibrium (Im et al., 2020; Li et al., 2022). Therefore, metabolic engineering strategies aimed at enhancing GABA yield must address not only the activation of biosynthetic routes but also the suppression or bypass of catabolic pathways. Xie et al. (2023) demonstrated that in *Lentilactobacillus curieae*, a GAD-deficient strain, GABA accumulation is dependent upon GABA-T activity, as genetic deletion abolished and complementation restored GABA production. They proposed that GABA-T may operate in reverse, though this contradicts the canonical GABA shunt where GABA-T strongly favors the degradative direction. Without flux data or redox/cofactor characterization, the directionality of this reaction remains speculative. The GABA-T-mediated route in LAB should therefore be regarded as a strain-specific metabolic curiosity rather than a universally conserved alternative pathway (Sharafi et al., 2019). This distinction is particularly important for industrial applications, as it underscores the need for strain-specific screening and tailored process design. This finding underscores the considerable metabolic diversity of GABA biosynthesis within the LAB phylogenetic group, necessitating strain-specific screening prior to industrial application. GABA-T-dominant strains may require neutral pH fermentation (H. Li et al., 2025) and precursor-directed metabolic engineering (Xie et al., 2023). Acid-shock and PLP-supplementation strategies are instead optimized for GAD-dependent producers. Integrating pathway identification with tailored process design will therefore be critical for maximizing GABA productivity across diverse LAB chassis. Fig. 3 provides a schematic overview of how the GABA shunt interconnects with the tricarboxylic acid (TCA) cycle and polyamine degradation pathways, highlighting the metabolic plasticity that underpins both GAD-dependent and GABA-T-mediated GABA production in LAB.

**Fig. 3:**
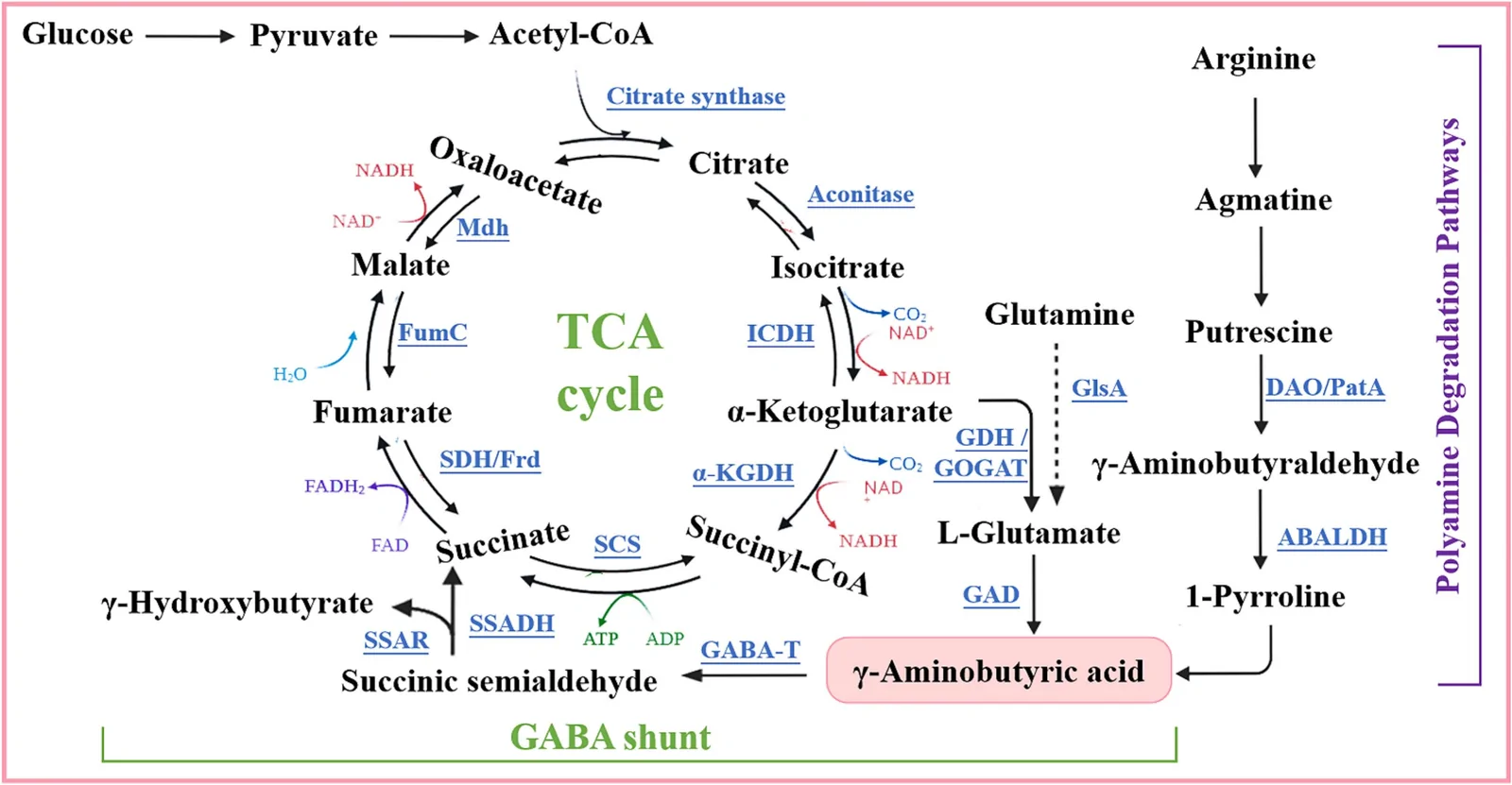
Integration of the GABA shunt, TCA cycle, and polyamine degradation pathways. The diagram illustrates key metabolic interconnections and the proposed, strain-specific involvement of GABA-T in the biosynthetic direction, which is hypothesized based on genetic evidence and requires further experimental validation of metabolic flux.

### 3.2 Integration and synergistic effects of metabolic networks

GABA synthesis in lactic acid bacteria is embedded within central carbon and nitrogen metabolism, forming a multi-pathway system with significant metabolic engineering potential. The gad operon encoding glutamate decarboxylase serves as the primary synthetic route, consuming intracellular glutamate and generating adenosine triphosphate (ATP) through the glutamate/GABA antiporter system. This process triggers dynamic depletion of the free glutamate pool, inducing compensatory metabolic reorganization (Wen & Bao, 2021). Glutamate deficiency activates the aspartic acid-glutamate axis, where increased aspartate aminotransferase activity promotes aspartic acid-glutamate conversion to maintain substrate homeostasis (Hertz & Rothman, 2017). Concurrently, glutamate depletion impacts arginine metabolism through upregulation of the arginase pathway, leading to ornithine and urea accumulation (Kim et al., 2017). These amino acid network interactions demonstrate that GABA biosynthesis cannot be optimized in isolation but requires systemic metabolic balancing and flux redistribution. PLP availability emerges as a central metabolic node that simultaneously governs GAD catalytic efficiency and the regenerative capacity of the glutamate supply network. Carbon and energy metabolism are intimately coupled with GABA production. GABA synthesis depends on ATP generated through glycolysis and is subject to feedback regulation by the synthetic pathway itself. Carbohydrate sources such as lactose trigger gad operon expression. Exogenous L-glutamate upregulates the phosphoenolpyruvate-sugar phosphotransferase system and glycolysis-related genes. Both effects feed into a positive feedback loop between carbon metabolism and GABA yield (Raethong et al., 2025; Shukla et al., 2018). This carbon-energy coupling extends to PLP metabolism, as PLP biosynthesis via the pyridoxal kinase pathway requires ATP and ribose-5-phosphate derived from the pentose phosphate pathway. Carbon flux partitioning between glycolysis and the pentose phosphate pathway indirectly modulates PLP supply, which in turn determines GAD activity and GABA output (Stander et al., 2025). GadC-mediated antiport generates ATP, part of which is diverted to PLP phosphorylation, which creates a reciprocal dependency. GABA synthesis consumes energy and cofactors, but it also regenerates the resources needed to sustain itself. Co-culture strategies can amplify this synergistic effect. For instance, *Levilactobacillus brevis* co-cultured with *Lactobacillus plantarum* achieves GABA yields of 224.69 mM through precursor provision and pyridoxal phosphate cofactor synthesis (Gomaa, 2015).

Future research should address several interconnected challenges. Enhancing GAD catalytic efficiency remains a priority, as kinetic characterization across diverse LAB strains reveals significant variation in catalytic efficiency and pH optima (Gu et al., 2021; Somasundaram et al., 2017). Redox balancing during high-yield fermentation presents another bottleneck; the metabolic burden of sustaining the glutamate/GABA antiporter often triggers premature growth arrest (Pannerchelvan et al., 2023; Tian et al., 2026). Genome-scale metabolic modeling offers a predictive framework for identifying cofactor regeneration and by-product diversion strategies. Systems-level integration through multi-omics and synthetic biology tools, such as LuxS/AI-2 quorum sensing (QS) systems, may further enable construction of chassis strains with maximized carbon flux toward GABA synthesis (Chai et al., 2025). A quantitative understanding of the production-consumption flux, including the contribution of GABA-T-mediated degradation under varying physiological conditions, remains limited and represents a key challenge for future research. Metabolic engineering strategies that simultaneously enhance biosynthetic capacity and minimize catabolic losses will be essential for achieving optimal GABA accumulation.

## 4 Screening and optimization of GABA-producing LAB

### 4.1 Strain screening

Traditional fermented foods such as yogurt, sauerkraut, pickles, and dairy products serve as natural reservoirs of microbial diversity, providing resources for screening lactic acid bacteria with specific functions. Indigenous microorganisms in these foods have undergone long-term natural domestication, possessing environmental stress resistance and efficient metabolic networks that make them valuable for discovering new GABA-producing strains (He et al., 2025). Certain LAB can perform lactic acid fermentation while activating the GAD system to convert glutamate to GABA (Zamanpour, Eraghi, et al., 2024). This represents both a mechanism for maintaining intracellular pH homeostasis and a route for obtaining this functional component. The reported GABA yields of LAB are summarized in Table 2. Since they were obtained under different experimental conditions and expressed in different units, these values should not be directly compared. In traditional fermented dairy products, bacterial genera such as *Lactiplantibacillus plantarum* and *Lactobacillus casei* have demonstrated GABA synthesis potential (Jena & Choudhury, 2024). Ayağ et al. Wu et al. (2025) isolated strains 134 and 255 of *Lactiplantibacillus plantarum* from Turkish handmade cheese that achieved a GABA yield of 626.36 ± 9.86 mg/L in MRS broth. This yield was significantly higher than common laboratory strains, highlighting the unique advantages of traditional resources for screening high-performance GABA-producing strains (Braga et al., 2024). This supports the view that direct screening from these ecological niches is an efficient strategy for obtaining high-yield wild-type strains without genetic engineering.

**Table 2:** Reported GABA-producing lactic acid bacteria.

Strains	Sources	Substrate/matrix	GABA yields	References
*Enterococcus avium* G-15	Carrot leaves	L-MSG in LB	115.7 ± 6.4 g/L	(Noda et al., 2024)
*B. adolescentis* IPLA60004/ IPLA60006	Belgium culture collection	MSG in MRS	0.896 mg/mL	(Tamés et al., 2023)
*L. pentosus* SS6	Fermented mulberry fruits	Mulberry leaf powder with sucrose, peptone, K_2_HPO_4,_ L-MSG	> 2.5 mg/mL	(Zhong et al., 2019)
*L. casei* ISL-7	Cheese	MgSO_4_·7H_2_O, KI, CaCl_2_, KH_2_PO_4_ in MRS	25.16 ± 0.13 g/L	(Ali et al., 2026)
*L. lactis* LEY7	Camel's milk	MSG in GM17	1.80 ± 0.10 mM	(Redruello et al., 2021)
*L. futsaii CS3*	Kung-Som	MSG in MRS	6.3 mg/mL	(Sanchart et al., 2016)
*L. brevis* F064A	Thai fermented sausage	MSG in MRS	2.85 ± 0.10 mg/mL	(Kanklai et al., 2020)
*L. brevis* F109-MD3	Korean kimchi	MSG in MRS	520 mmol/L	(Liu et al., 2022)
*L. brevis RK03*	Red bigeye fish	Glucose, yeast extract, CaCO_3_, MnSO_4_, and Tween 80, PLP, MSG	62.523 mg/L	(Wu et al., 2018)
*L. brevis* NM101–1	Egyptian dairy products	MSG, PLP in MRS	168.58 mM	(Gomaa, 2015)
*L. plantarum* DSM749			140.69 mM	
*L. plantarum* EJ2014	Rice bran	MSG, yeast extract, dextrose	19.8 g/L	(Park et al., 2021)
*L. plantarum* Taj-Apis362	Honey stomach	MSG in MRS	7.15 mM	(Tajabadi et al., 2015)
*L. plantarum* MCM4	Korean kimchi	Soy protein hydrolysate, whey	0.365 mg /mL (co-culture with *Lc. lactis* subsp. *Lactis*)	(Karimian et al., 2020)
*L. plantarum* Lp3	Korean kimchi	Glucose, yeast extract, Ca^2+^	3.74 mg/mL	(Wang, Wang, Nan, et al., 2024)
*L. plantarum* JY7	Fish intestine	MSG, PLP in MRS	1.0014 mg/mL	(Yuan et al., 2026)
*L. plantarum* 134/255	Turkish handmade cheese	MSG in MRS	626.36 mg/L	(Ayağ et al., 2025)
*L. plantarum* NDC75017	China traditional fermented dairy products	L-MSG, PLP in MRS	314.56 mg/100 g	(Shan et al., 2015)
*L. plantarum* H64	Hop	MSG, PLP, bread slurries	521 ± 10 mg/L	(Verni et al., 2022)

**Note:** GABA yields are reported as originally published and are not directly comparable across studies due to differences in units (g/L, mg/100 mL, mg/kg, mM, μg/mL, mg/mL), fermentation conditions, detection methods, and strain backgrounds. Readers should exercise caution when interpreting cross-study comparisons.

As illustrated in Fig. 4, the workflow for screening GABA-producing LAB encompasses the complete pipeline from sample collection to advanced multi-omics profiling. Samples are first enriched under acidic or glutamate-supplemented conditions to select GABA-producing strains (GAD-positive). High-throughput phenotypic screening then follows with pH indicators or thin-layer chromatography (TLC). Promising candidates are quantified by HPLC and screened genetically to confirm *gad* operon integrity. Such integrity reliably predicts yield potential, and strains with complete *gadB* and *gadC* can reportedly achieve up to 2.3-fold higher production (Langa et al., 2024). Beyond this standard pipeline, metabolomics-guided screening using GABA transaminase inhibitors has achieved higher intracellular GABA accumulation by blocking catabolism (Surachat et al., 2021), while transcriptomic profiling of GAD regulatory networks enables rational strain selection beyond simple gene presence–absence assays. These strategic variations shift screening from empirical observation toward systems-level design, though their broader adoption remains limited by cost and technical expertise. Table 3 presents a comparative overview of commonly employed GABA detection methods, summarizing their underlying principles, sensitivity, key advantages, and principal limitations. The choice depends on research stage and throughput requirements. In preliminary screening, substrate-specific methods based on pH indicators such as bromocresol purple offer high throughput but are susceptible to interference from other acid-producing pathways (Devi et al., 2023; Heru Nurcahyo et al., 2019). TLC enables rapid qualitative assessment by comparing retention factor (Rf) values of GABA and monosodium glutamate. The method is simple to operate and cost-effective, its limited resolution nevertheless restricts application to preliminary identification rather than accurate quantification (Duyen et al., 2023; Rehman et al., 2023). For precise quantification, HPLC were reported detection limits span 0.01 μg/mL to roughly 1.0 μg/mL and vary with the derivatization reagent, detector type, and analytical conditions. HPLC also delivers excellent reproducibility across diverse matrices such as brown rice, beans, fermented lentils, melon, and tea leaves (Barba et al., 2015; Hayat et al., 2014; Li et al., 2010; Mockus et al., 2023; Toyoizumi et al., 2020; Zhang, Guo, et al., 2023). Its primary drawbacks are reagent instability and time-consuming derivatization pretreatment. Ultra-performance liquid chromatography (UPLC) offers superior efficiency and sensitivity but similarly requires cumbersome derivatization steps (Paris et al., 2011). Other quantification approaches, such as enzyme-based methods allow simultaneous analysis of multiple samples, though they require an early aldehyde separation step (Kittibunchakul et al., 2017; Himi, 2024). Amino acid analysers, which provide high specificity and good separation from other amino acids but are limited by high cost and extended analysis time (Ma, Wang, Yang, et al., 2022; Ma, Wang, Zhou, et al., 2022; Xu et al., 2010; Yoon et al., 2017). Colorimetric methods are simple, fast, and well suited to large-scale screening, but are sensitive to reagent dosage, coloring time, and light exposure (Jinnarak & Teerasong, 2016). Direct real-time mass spectrometry allows analysis without chromatographic separation or derivatization, yet the requirement for specialized instrumentation limits its widespread adoption (Li et al., 2019). Nuclear magnetic resonance (NMR) can confirm structural identity, but its high cost prevents large-scale application. In practice, laboratories typically adopt a tiered strategy. Preliminary screening is performed using TLC or colorimetric methods to maximize throughput. Precise quantification then follows using HPLC or UPLC for accuracy. The final choice of method depends on sample throughput, matrix complexity, accuracy requirements, and equipment availability (Tiwari et al., 2018).

**Fig. 4:**
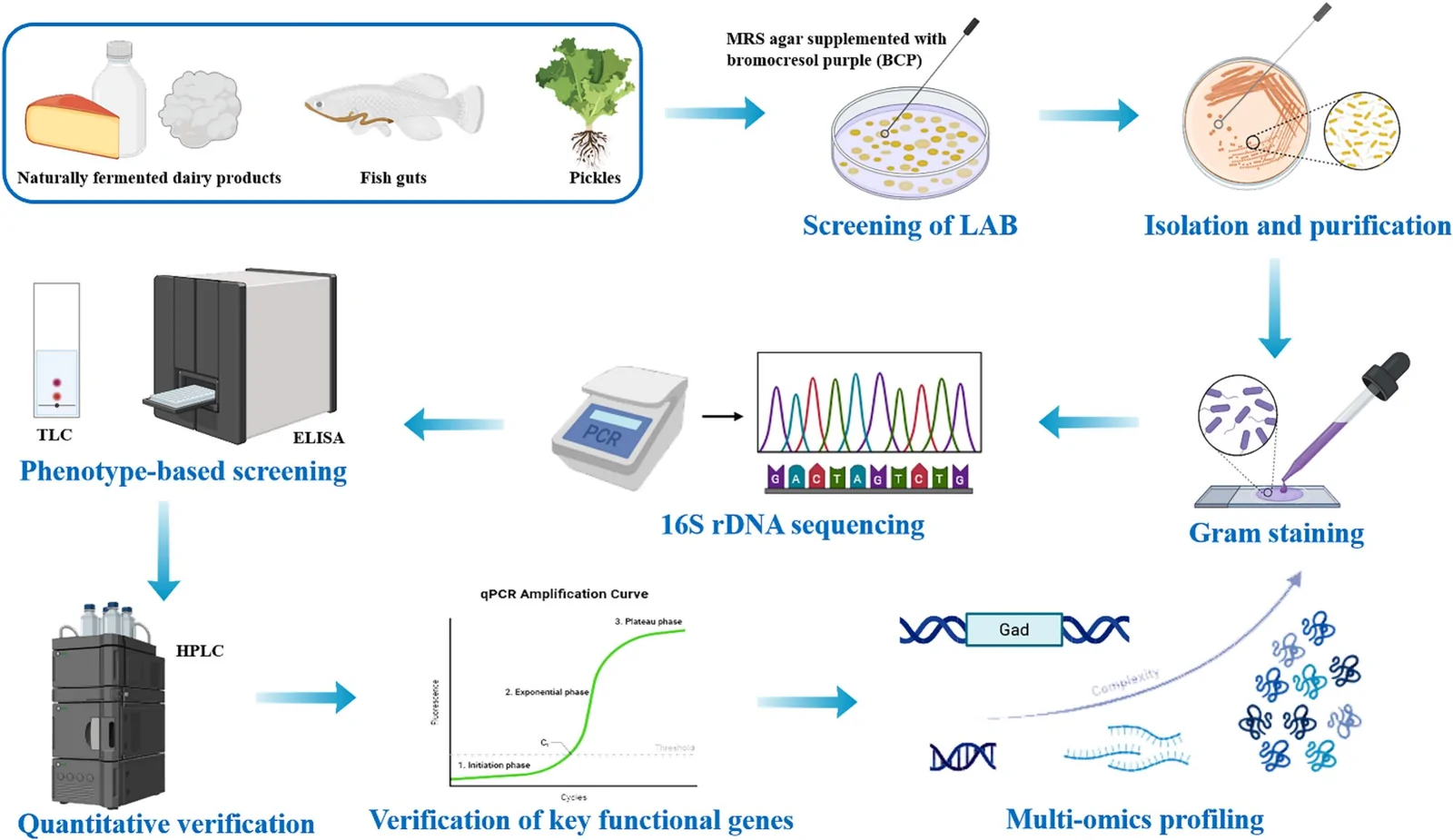
The workflow for screening of high-yield GABA-producing lactic acid bacteria. This begins with isolation and purification of candidate strains, proceeds to phenotypic screening using BCP indicator and TLC, followed by quantitative verification, then genotypic confirmation targeting the integrity of the gad operon, and multi-omics profiling (metabolomics/transcriptomics) for mechanistic elucidation of GABA metabolic networks.

**Table 3:** Methods for GABA content determination and their characteristics.

Method	Advantages	Limitations	Application	References
High-performance liquid chromatography (HPLC)	Widely used, established method	Reagents unstable, time-consuming process	Pakistani brown rice	(Hayat et al., 2014)
			Beans	(Barba et al., 2015; Li et al., 2010)
			Fermented lentils	(Mockus et al., 2023)
			Greenhouse muskmelon	(Toyoizumi et al., 2020)
			Tea leaves	(Zhang et al., 2019)
Enzyme-based method	Suitable for simultaneous analysis of multiple samples	Requires early aldehyde separation step	Thai brown rice	(Kittibunchakul et al., 2021)
			Wheat	(Himi, 2024)
Amino acid analyzer	High specificity for GABA, good separation from other amino acids	Expensive, time-consuming	white tea	(Ma, Wang, Zhou, et al., 2022)
			spinach	(Yoon et al., 2017)
			Oats	(Xu et al., 2010)
Calorimetric method	Simple, fast, suitable for large-scale screening	Sensitive to reagent dosage, coloring time, and light exposure	Dietary supplements	(Jinnarak & Teerasong, 2016)
Ultra Performance Liquid Chromatography (UPLC)	High efficiency and sensitivity	Requires cumbersome derivatization	Vigna radiate	(Paris et al., 2011)
Thin layer chromatography (TLC)	High precision and good linearity	Not ideal for complex sample matrices	Sumbawa mare milk	(Nursini et al., 2022)
			Rice	(Bai et al., 2025)
Direct real-time mass spectrometry	No chromatographic separation or derivatization needed; direct analysis	Requires specialized instrumentation	Fermentation beans/ germinated barley	(Li et al., 2019)

Genetic analysis has shifted screening from empirical phenotypic observation toward rational genotypic identification (Lyu et al., 2018). According to Langa et al. (2024), lactic acid bacteria with a complete *gad* operon produce 2.3 times more GABA than wild-type strains. This directly shows that the intact gad gene cluster is a crucial molecular marker for high-yield strain evaluation. Metabolomics and transcriptomics can reveal regulatory networks in GABA synthesis, identifying targets for metabolic engineering (Surachat et al., 2021). However, existing research largely concentrates on individual strain capacity, with incomplete understanding of metabolic networks in complex fermentation systems and regulatory mechanisms of the GAD system. Exploring the combination of traditional strains with modern gene editing technologies may yield breakthroughs in screening and optimization. Emerging artificial intelligence (AI) technologies have transformative potential for accelerating GABA-producer discovery. For example, machine learning algorithms can be trained on genomic and phenotypic datasets. They predict GAD activity and GABA yield directly from sequence data before any laboratory validation. This approach significantly reduces the screening burden (Shi et al., 2026). Deep learning models can identify promoter-gene pairs that boost metabolic flux toward target compounds. Convolutional neural networks also help predict off-target effects in CRISPR-based strain engineering (Priyadharshini et al., 2025). High-throughput microtiter plate screening can be integrated with AI-guided analytical pipelines. This integration enables automated evaluation of fermentative potential across thousands of variants without operator intervention (Cui, Qi, et al., 2025). These computational methods complement empirical screening rather than replace it. While these computational methods have shown promise in other microbial systems, their direct application to high throughput screening of GABA producing lactic acid bacteria remains to be validated.

### 4.2 Effective strategies for increasing GABA production by LAB

After obtaining lactic acid bacterial strains capable of synthesizing GABA, the core question becomes how to systematically enhance their yield. Current optimization strategies present a diverse technological landscape, ranging from direct genetic intervention to fine-tuning of the external environment, and together form a complete system that operates from inside to outside the cell and from single-target to systemic regulation (Fig. 5). The effectiveness and industrial applicability of these strategies vary considerably. Genetic and metabolic engineering approaches deliver the highest yield improvements, but require substantial R&D investment and face regulatory hurdles for food-grade applications. Fermentation process optimization offers moderate yield gains at relatively low cost and is readily scalable, though its effectiveness is bounded by the inherent capacity of the host strain (Wang et al., 2018). Physical and chemical stimulation methods provide modest enhancements with simple implementation, yet suffer from poor reproducibility and potential cytotoxicity at industrial scale (Sidooski et al., 2019). Synthetic microbiota strategies leverage interspecies synergies for substantial yield increases, but present significant challenges in process controllability and strain compatibility. These trade-offs suggest that no single strategy is universally optimal. Instead, integrated multi-level approaches are the most promising route for industrial translation. Such approaches combine genetic foundation with process refinement and ecological augmentation. Genetics and metabolic engineering provide the most fundamental means of modification, aiming to strengthen the synthetic capacity of strains at the source. For example, atmospheric and room temperature plasma mutagenesis increased the GABA yield of *Lactobacillus brevis* from 2.32 g/L to 3.86 g/L, with the mutant strain UV5–8 showing 5.4-fold upregulation of *gad* encoding glutamate decarboxylase (Zeng et al., 2025). Blocking competitive pathways and strengthening precursor supply constitute synergistic strategies beyond enhancing core enzyme activity. Yu et al. (2019) combined knockout of *gabT*, *gabP*, and *puuE* with overexpression of *gadA*, *gadB*, and *gadC* in the engineered strain *E. coli* ZGK12-TPE/pTrc99a-gadABC to achieve 19.79 g/L GABA, offering a conceptual framework for analogous metabolic engineering in lactic acid bacteria. However, gene editing may reduce genetic stability, and incomplete understanding of metabolic networks can limit pathway optimization (Peng & Liang, 2020). From a food-safety and regulatory perspective, the application of genetically modified LAB in food products faces substantial hurdles. Regulatory frameworks for GMO-based foods vary across jurisdictions, and consumer acceptance remains a significant barrier in markets favoring non-GMO products (Fitsum et al., 2025). Safety concerns include horizontal gene transfer and the need for industrial containment (Spano et al., 2010). These challenges have motivated non-GMO alternatives such as adaptive laboratory evolution, strain selection, process optimization, and co-culture strategies (Ayağ et al., 2025; Gong et al., 2023; Tan, Zhang, Liu, Shang, et al., 2024). For instance, adaptive laboratory evolution has been applied to *Escherichia coli* W, achieving a 3.9-fold increase in GABA titer through iterative selection under progressive metabolic stress (Kim et al., 2022), offering a conceptual framework for future strain improvement in LAB. While genetic engineering remains a powerful tool, its practical application in food systems must be weighed against regulatory, safety, and consumer considerations.

**Fig. 5:**
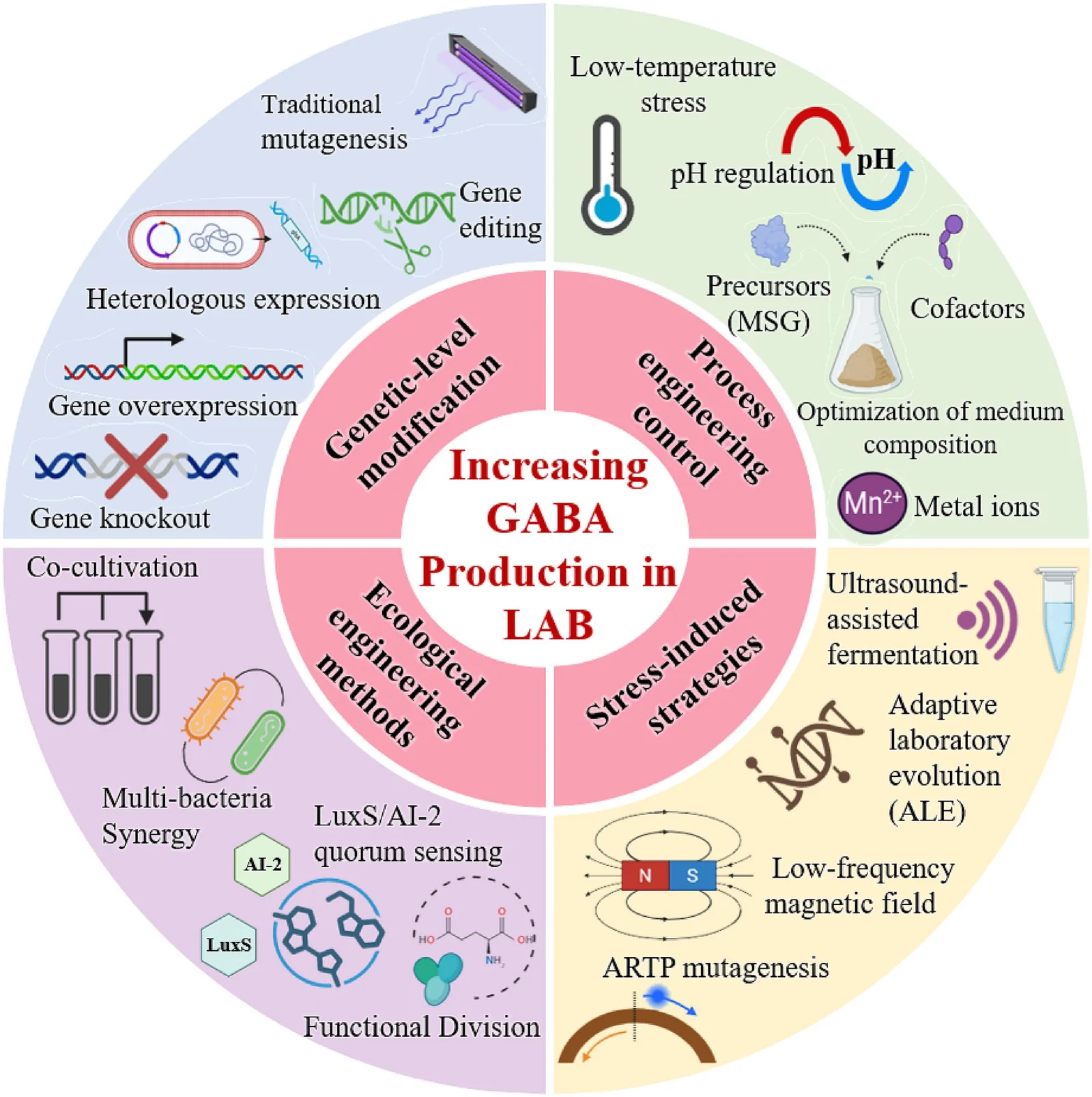
Multidimensional strategies for enhancing GABA production by lactic acid bacteria. The four strategies include genetic and metabolic engineering, fermentation process optimization, physical and chemical stimulation, and synthetic microbial communities, forming a complete technical system from intracellular genetic modification to extracellular ecological synergy.

Fermentation process optimization, as a non-genetic approach, is more readily applicable to industrial scale-up. Optimizing the culture medium by adding monosodium glutamate and PLP can increase yield from 165.1 mg/L to 211.4 mg/L (Rezaei et al., 2023). The optimal values for fermentation parameters vary considerably across LAB strains and production systems. pH optima for GAD activity typically range from 4.0 to 5.5 depending on the strain. Temperature optima generally fall between 30 °C and 37 °C. Glutamate supplementation levels are commonly reported between 1% and 3% (*w*/*v*). These ranges are strain-dependent, and optimal conditions must therefore be determined empirically for each specific system. The interactions among multiple factors, such as the coupling between pH control and substrate feeding, often influence overall yield more significantly than any single parameter in isolation. Dynamic strategies such as two-stage pH control can enhance enzyme activity and cell tolerance, enabling *Lactobacillus hilgardii* GZ2 to achieve 229.3 g/L (Gong et al., 2023). Such strategies are constrained by inherent strain capacity but offer yield increases at relatively low cost. Physical fields and chemical stimuli have demonstrated regulatory potential by inducing stress responses. The most widely investigated approaches include ultrasound treatment and low-frequency magnetic fields, which operate through distinct biophysical mechanisms. Ultrasound treatment generates mechanical vibrations and cavitation effects that transiently increase cell membrane permeability, facilitating enhanced substrate uptake and product release (Shokri et al., 2021). Low-frequency magnetic fields are thought to modulate cellular metabolism through free radical signaling pathways and altered ion flux across membranes, which may upregulate gad gene expression (Poojary et al., 2017; Zablotskii et al., 2025). Other physical methods such as high-pressure processing, pulsed electric fields, and microwave treatment have not been extensively studied for GABA-producing LAB, and their direct application remains limited in the current literature. As a mutation-based approach rather than a physical stimulation method, atmospheric and room temperature plasma (ARTP) mutagenesis has been shown to significantly increase the GABA yield of *Lactobacillus brevis* (Yogeswara, Kittibunchakul, et al., 2020; Yogeswara, Maneerat, & Haltrich, 2020). In chemical treatments, Mn^2+^ and PLP can enhance enzyme activity, but concentrations must be controlled to avoid cytotoxicity and manage costs (Sun et al., 2022).

Synthetic microbiota strategies move beyond single-strain optimization to leverage microbial interactions (Diez-Gutiérrez et al., 2022; Jo et al., 2019). Unlike spontaneous fermentation, which relies on undefined microbial consortia, synthetic co-culture employs well-characterized strains with complementary metabolic roles. Tan, Zhang, Liu, Xiang, et al. (2024) found that the cell-free supernatant of *Lactiplantibacillus plantarum* BC112 could significantly promote GABA yield in *Enterococcus faecium* AB157, with a maximum increase of 4.6-fold. This cross-feeding mechanism involves metabolic intermediates or signaling molecules produced by L. *plantarum* BC112 that stimulate GABA synthesis in *E. faecium* AB157. Furthermore, Tan, Zhang, Liu, Shang, et al. (2024) co-cultured *E. faecium* AB157 with *Saccharomyces cerevisiae* SC125 at a ratio of 4:1 and supplemented with 12 g/L MSG, achieving a yield of 6.57 g/L. *S. cerevisiae* SC125 likely supports *E. faecium* AB157 through substrate conversion or cofactor provision. Interspecific relationships such as cross-feeding represent an alternative avenue for driving GABA synthesis, though controllability presents challenges for industrial application. The optimization of GABA yield now relies on a multi-level technological system. Its components include genetic modification as the foundation, process optimization for efficient production, physical and chemical stimulation for fine-tuning, and ecological strategies for maximizing overall efficiency (Wu et al., 2022). However, all strategies face challenges in industrial scale-up, including R&D costs, equipment investment, and process controllability. Artificial intelligence is increasingly applied to fermentation process optimization in the broader field of biotechnology. Reinforcement learning algorithms have been demonstrated to dynamically adjust pH, temperature, and substrate feeding in real-time based on sensor data, improving yield consistency and reducing batch failure rates (Wang, Zeng, et al., 2025). For GABA production specifically, AI-driven models could potentially use multi-omics data to predict optimal induction conditions for gad operon expression and simultaneously monitor biogenic amine formation as a constraint, though such integrated applications have not yet been reported for GABA-producing LAB. Process optimization should balance GAD activity against histidine decarboxylase suppression, with dual monitoring of GABA titer and biogenic amine levels as standard practice (Dankar et al., 2025).

## 5 Applications of GABA-producing LAB in the food industry

### 5.1 Fermented dairy products

GABA-producing lactic acid bacteria are a crucial link between food processing and health benefits. They achieve multiple benefits through biotransformation in diverse food matrices. These benefits include flavor optimization, shelf-life extension, and potential health benefits such as mood regulation and cardiovascular health support that have been investigated primarily in preclinical studies (Fig. 6). Fermented dairy products represent an ideal delivery vehicle for GABA, offering the organic integration of food processing, in situ functional component biosynthesis, and systemic health benefits. Table 4 highlights that processed dairy products such as tomato juice fermented by *Lactiplantibacillus plantarum* KB1253 achieve 41.0 ± 1.1 mM GABA, while bread products with L. *plantarum* H64 reach 30 μg/g. Soy yogurt fermented by L. *plantarum* GA30 achieves specific modulation of beneficial gut microbiota (*Lactobacillus, Bifidobacterium*, and *Akkermansia muciniphila*), delivering multiple health benefits from neuroregulation to microbiota modulation (Weng et al., 2023). *L. plantarum* KCTC 3103 utilizes its GAD system to consume H^+^ during metabolism, naturally regulating pH while promoting continuous glutamate-to-GABA conversion (Kim et al., 2021). Fan, Jiang, et al. (2023) and Fan, Yu, et al. (2023) developed a novel flavored yogurt using *Levilactobacillus brevis* CGMCC 1.5954, achieving a GABA content of 147.36 mg/100 mL. Controlled fermentation at 37 °C with 2% (*w*/*v*) monosodium glutamate improved the strain's performance. This improvement highlights that precursor availability and pH control are crucial for GABA yield in industrial yogurt production. *L. rhamnosus* GG, characterized by an adhesion index ≥8, effectively prolongs GABA local action time and enhances bioavailability, facilitating sustained release and absorption to regulate gut-brain axis homeostasis (Ganguli et al., 2015). However, the GABA-producing capacity of this strain remains modest, highlighting the trade-off between colonization ability and metabolic output that strain selection must navigate.

**Fig. 6:**
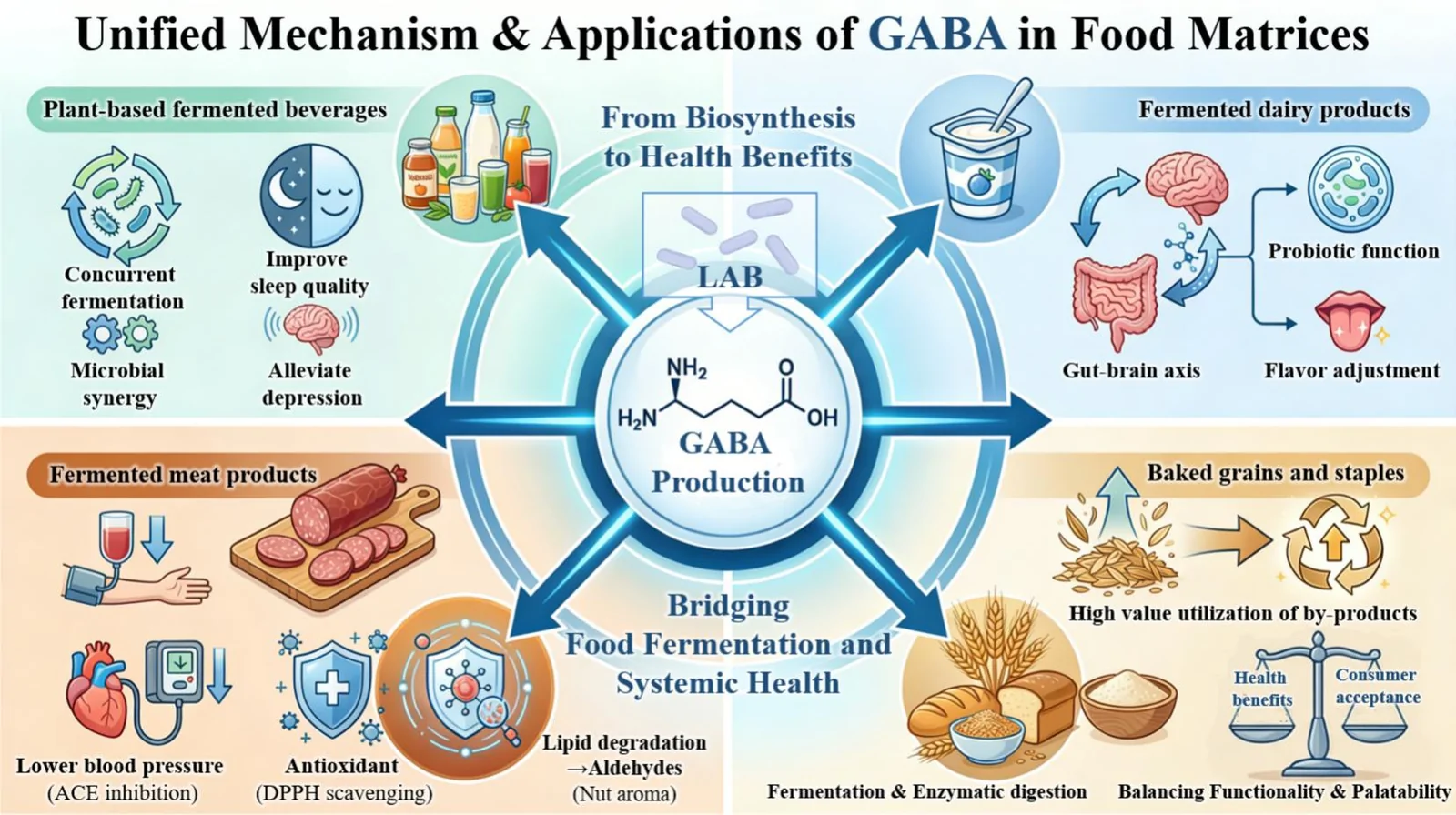
GABA-producing lactic acid bacteria may contribute to flavor improvement, shelf-life extension, and potential health benefits, including mood regulation, stress relief, and cardiovascular health support. Effects shown are based primarily on preclinical studies; human clinical validation is needed.

**Table 4:** Natural and processed foods containing GABA.

Source	Type	Treatment	GABA content	References
Natural	European gooseberry	–	13.2 mg/100 g	(Pencheva et al., 2022)
	Blueberry		8.6 mg/100 g	
	Potato		44.9 mg/100 g	
	Broccoli		12.88 mg/100 g	
	Red beet		18.8 mg/100 g	
	Parsley		28.2 mg/100 g	
	Dwarf elderberry		11.1 mg/100 g	
	Lentil		10.4 mg/100 g	
	Quinoa		10.5 mg/100 g	
	Yellow tomato		36.8 mg/100 g	
	Germinated brown rice	Ultrasonic-assisted cellulase pretreatment	46.46 mg/100 g	(Chamsai & Wanyo, 2025)
	Mung beans	Heat and relative humidity (HRH) treatment	102.32 mg/100 g	(Ma, Wang, Yang, et al., 2022)
	Mixed HRH-MB-wheat flour	–	21.60 mg/100 g	
	Chinese yam tubers	Carbon dioxide	1.25 ± 0.08 mg/g	(Wang, Chen, et al., 2025; Wang, Zeng, et al., 2025)
		Nitrogen	0.31 ± 0.03 mg/g	
		Vacuum	0.39 ± 0.02 mg/g	
		Water	0.73 ± 0.04 mg/g	
Processed	Tomato juice	*Lactiplantibacillus plantarum* KB1253	41.0 ± 1.1 mM	(Nakatani et al., 2022)
	Wheat bread	*Lactiplantibacillus plantarum* H64 and *Saccharomyces boulardii* CNCM I-745	321.58 μg/mL	(Zhang, Yousefvand, et al., 2025)
	Rice bread	*Saccharomyces cerevisiae*	89.7 mg/100 g	(Zhang, Kitaori, & Lai, 2025)
	Germinated brown rice sourdough bread	*Lactobacillus sakei*; *Lactobacillus sanfranciscensis*	About 6.5 mg/100 g	(Sobhanian et al., 2025)
	Sourdough bread	*L. farciminis* A11	39 mg/kg	(Venturi et al., 2019)
	Legume-based beverage	*Lactobacillus plantarum* C9S2	2262.21 ± 219.4 mg/L	(Montagano et al., 2025)
	Fermented quinoa	*Lactobacillus plantarum* NO.2	74.2 mg/100 g	(Zhang, Zhang, et al., 2022)
	Soy sauce	*Bacillus cereus* KBC; *Tetragenococcus halophilus* KBC; *Aspergillus oryzae* NSK	239.08 mg/L	(Shin Yee et al., 2024)
	White brined cheese	*L. pentosus* C22	16.5 mg/100 g	(Mohammadzadeh et al., 2025)
	Fermented mulberry leaf powder	*Lactobacillus pentosus* SS6	54.96 ± 0.02 mg/g	(Zhong et al., 2019)
	Fermented mulberry Juice	*Levilactobacillus brevis* F064A	3.31 ± 0.06 mg/mL	(Kanklai et al., 2020)
	Fermented rice flour	*Levilactobacillus brevis* TISTR 860	21.80 mg/100 g	(Chittrakhani et al., 2022)
	Fermented water dropwort	*Leuconostoc mesenteroides* SM; *Lactobacillus plantarum* K154	10 mg/mL	(Kwon et al., 2016)

**Note:** GABA contents are reported in original units as published; direct cross-study comparison is not recommended due to inconsistent units and experimental conditions.

The industrial production of GABA-enriched dairy requires optimization of fermentation parameters (glutamate concentration 1–3%, pH 4.0–5.5, temperature 30–37 °C), with two-stage pH control strategies improving yields by up to 40% (Gong et al., 2023). PLP supplementation (50–400 μM) is often necessary for maximal GAD activity, though cost-benefit analysis is required (Li et al., 2022). GABA is generally stable during refrigerated storage but can degrade 15–30% upon thermal processing, necessitating cold-chain strategies or microencapsulation (Ma, Wang, Yang, et al., 2022; Ma, Wang, Zhou, et al., 2022; Misra et al., 2025). Its stability in dairy matrices is further influenced by pH and interactions with milk proteins, which may affect GABA retention during processing and storage. GABA itself is tasteless, but residual glutamate may alter umami intensity. Co-fermentation with adjunct cultures can balance flavor profiles, and consumer acceptance remains favorable (>7.0/9-point scale) when GABA is health-positioned (Fan, Jiang, et al., 2023; Fan, Yu, et al., 2023). The antihypertensive effects of GABA-enriched dairy (10 mmHg systolic blood pressure reduction) are supported by animal experimental data (spontaneously hypertensive rats) (Chen et al., 2018). Sleep-improving effects have been evaluated in human subjects consuming GABA-rich fermented milk (33.33 mg/kg) (Yu et al., 2020), while anxiolytic and memory-enhancing effects have been demonstrated in animal models (Fan et al., 2025). Direct clinical human trials validating mood regulation outcomes from GABA-enriched dairy consumption remain limited, representing a priority for future research to substantiate health claims in regulated markets.

### 5.2 Plant-based fermented beverages

Plant-based matrices present unique challenges for GABA enrichment due to protein complexity and glutamate deficiency, yet synthetic microbial community strategies offer effective solutions. Table 4 shows that legume-based beverages with L. *plantarum* C9S2 achieve 2262.21 mg/L GABA. Brown rice milk fermented by L. *pentosus* 9D3 achieves 14.3 mg/100 mL GABA content while maintaining viable cell counts exceeding 8.6 log CFU/mL alongside high antioxidant activity (Kittibunchakul et al., 2021). Co-fermentation using L. *brevis* 877G (high protease activity) and L. *sakei* 795G (efficient GABA conversion) systematically addresses plant matrix limitations through metabolic division of labor, decoupling protein hydrolysis from GABA synthesis to achieve 22.51 mM production (Seo et al., 2013). Hong Le et al. (2021) demonstrated that precursor liberation is the critical rate-limiting step in flavor development within plant-based systems. Plant substrates have lower free glutamate content, typically 0.5–2.0 mg/g, compared to 10–20 mg/g in dairy. To match dairy-based GABA yields, either exogenous MSG (1–3%) or pre-hydrolysis strategies are required. Fermentation temperature and pH control also play key roles. The recommended temperature is 32–37 °C. The pH is maintained at 5.0–6.0 during early growth and then lowered to 4.0–4.5 for GAD activation. In functional fermented food and dairy food product, these optimizations improve conversion efficiency by 25–35% (Seo et al., 2013).

Plant-based GABA beverages face stabilization issues such as protein precipitation during acidification and beany off-flavor retention in soy matrices (Nedele et al., 2021). Homogenization at 20–30 MPa before fermentation and the addition of stabilizers like pectin or carrageenan can improve suspension stability and mouthfeel (Heiden-Hecht et al., 2023). During refrigerated storage, GABA retention in plant matrices is comparable to dairy. The loss is under 10% over 21 days, but thermal pasteurization leads to 20–25% degradation. Therefore, high-pressure processing or aseptic cold filling might be better for premium products (Kullananant et al., 2020). For industrial scale-up, a key economic variable is the cost-effectiveness of enzymatic pre-hydrolysis versus direct MSG supplementation. The fermented adzuki bean milk (241.30 μg/mL GABA) alleviated depressive-like symptoms via GABA_B_-cAMP-PKA-CREB pathway activation in mouse models (Wu et al., 2021). Clinical human trials specifically evaluating plant-based GABA beverages for sleep or mood outcomes are currently absent. Existing studies focus on physicochemical characterization and microbiological safety (Kittibunchakul et al., 2021). The translation of preclinical findings to human health claims requires rigorous clinical validation, which remains a priority for regulatory approval in functional food markets.

### 5.3 Fermented meat products

GABA-producing LAB introduce functional attributes into traditional fermented meats while preserving authentic flavors and processing characteristics, and this category has garnered increasing attention for its potential to transform conventional cured products into functional foods. Traditional Thai fermented sausage “Sai Krok Esan” and Turkish sucuk utilize indigenous LAB to enhance product value through dual mechanisms of flavor development and bioactive compound generation (Kamiloğlu, 2022; Vichitphan et al., 2013). *L. sakei* CRL1756 converts meat-derived glutamate into GABA while simultaneously producing hexanal and other aldehyde compounds through lipid degradation, imparting distinctive nutty aromas (Montanari et al., 2018). The balance between desirable and undesirable flavors ultimately depends on the interplay of amino acid catabolism, lipid oxidation, and organic acid production during fermentation. The antioxidant properties of GABA (DPPH IC₅₀ 0.8 mg/mL) effectively inhibit lipid oxidation, extending shelf life while maintaining traditional quality attributes (Zareian et al., 2020).

The application of GABA-producing LAB in meat matrices involves distinct technological considerations. Meat proteins provide abundant endogenous glutamate (5–8 mg/g), which reduces the need for exogenous precursor supplementation compared to plant-based systems. However, pH control remains critical because meat fermentation typically operates at higher pH ranges (5.0–5.8) than dairy, and this higher pH can limit GAD activity (Zhao et al., 2016). Starter culture selection must balance GABA productivity with proteolytic and lipolytic activities essential for typical fermented meat flavor development. Temperature control presents a trade-off, as traditional fermentation uses 20–25 °C whereas GABA optimization requires 30–37 °C, affecting both microbial growth and product texture. Post-fermentation handling also affects GABA retention, since drying and smoking processes common in traditional meat products can expose GABA to oxidative degradation. Modified atmosphere packaging or antioxidant incorporation may be necessary to maintain stable GABA content throughout shelf life (Thanh et al., 2025). While these results demonstrate promising antihypertensive potential, human clinical trials for GABA-enriched fermented meats are absent, limiting direct health claim substantiation. Extrapolating animal data to human cardiovascular outcomes requires further investigation, especially regarding GABA bioavailability from meat matrices and optimal dosing regimens for blood pressure management. Consumer acceptance studies are also scarce, but GABA's tasteless nature suggests minimal sensory interference, and the health-positioning of traditional fermented meats may align with growing consumer demand for functional protein sources.

### 5.4 Baked goods and fermented cereals

Cereal matrices and processing by-products are being re-endowed with functional value through GABA-producing LAB, aligning with sustainable food system development and addressing the underutilization of nutrient-rich side streams. Table 4 highlights that germinated brown rice bread fermented by L. *sakei* and L. *sanfranciscensis* achieves approximately 6.5 mg/100 g GABA content with favorable consumer acceptance (Sobhanian et al., 2025), while sourdough bread with multiple *Lactobacillus* species reaches up to 39 mg/kg (Venturi et al., 2019). Rice germ (a by-product typically discarded due to lipid oxidation susceptibility) exhibits significant neuroprotective functions after L. *brevis* BJ20 fermentation, increasing cell viability by 35% and reducing lactate dehydrogenase release by 58% in corticosterone-stressed neuronal models (Hyun et al., 2025). Spice addition synergistically enhances GABA accumulation while imparting unique flavors (Hisaki et al., 2024), and amaranth-enriched sourdough improves both GABA content and antioxidant capacity.

Germination and fermentation are often combined to maximize GABA content. Germination activates endogenous glutamate decarboxylase and increases free glutamate availability, subsequent LAB fermentation then converts this pool to GABA (Li et al., 2021). However, baking temperatures (180–220 °C) pose a significant threat to GABA stability. Studies indicate 40–60% degradation during conventional baking (Polak et al., 2021). The extent of degradation depends on baking temperature, time, and the water activity of the dough, with higher temperatures and longer processing times accelerating GABA loss. This suggests that post-baking application of GABA-enriched spreads or cold-fermented cereal products may offer better functional retention. The high water activity in bread and baked goods also supports microbial growth during storage. This necessitates modified atmosphere packaging or clean-label preservatives to maintain both GABA content and food safety throughout shelf life (Qian et al., 2021). Multi-objective optimization balancing GABA yield, sensory quality, and processing characteristics remains critical for successful market introduction of GABA-enriched staple foods. The integration of upcycled by-products such as rice germ and bran into GABA-enriched formulations offers both economic and environmental advantages that align with circular economy principles in food production.

## 6 Conclusion

This review systematically examines GABA-producing LAB, focusing on GAD system mechanisms, strain optimization, and applications in dairy, plant-based, meat, and cereal products. The GAD system functions both in acid resistance and GABA biosynthesis, enabling efficient production in food-grade organisms, and the integration of screening, metabolic engineering, and process optimization has significantly improved GABA yield and conversion efficiency. In food systems, GABA-producing LAB have demonstrated considerable promise in preclinical models for health benefits such as hypotensive, antioxidant, and microbiota modulation effects, but human clinical data remain limited and well-controlled intervention studies are critically needed. Additional limitations include poor translation from animal models, unclear GABA bioavailability, difficulty in distinguishing GABA-specific effects from synergistic metabolites, biphasic immune responses, and trade-offs between GABA production and biogenic amine formation. Industrial challenges involve incomplete precursor conversion, poor GABA stability during processing and storage, and genetic instability in engineered strains. The sensory quality of GABA-enriched products is shaped by a complex network of metabolic by-products, and further mechanistic studies on flavor formation would benefit the development of consumer-acceptable functional foods. Future work should focus on elucidating putative non-GAD pathways and strain adaptation, developing genetically stable high-producers, expanding into plant-based systems and waste valorization, integrating sensory and functional design, incorporating amine screening in strain selection, and implementing dual-analysis methods for GABA and amine monitoring. The convergence of food science and microbial engineering, including emerging computational tools, can build a complete value chain from GABA synthesis to food functionalization, thereby advancing nutrition, innovation, and sustainability.

## Declaration of generative AI in scientific writing

Generative AI and AI-assisted technologies should only be used in the writing process to improve the readability and language of the manuscript.

## CRediT authorship contribution statement

**Hui Zhou:** Visualization. **Wenying Yuan:** Writing – original draft, Visualization, Methodology, Data curation, Conceptualization. **JiaoJiao Hu:** Investigation. **Mengyao Liu:** Methodology. **Jie Luo:** Writing – review & editing, Formal analysis. **Baokun Li:** Data curation. **Xiankang Fan:** Project administration, Funding acquisition, Conceptualization.

## Declaration of competing interest

The authors declare that they have no known competing financial interests or personal relationships that could have appeared to influence the work reported in this paper.

## Data Availability

Data will be made available on request.
